# Multi-objective optimization of custom implant abutment design for enhanced bone remodeling in single-crown implants using 3D finite element analysis

**DOI:** 10.1038/s41598-024-66807-2

**Published:** 2024-07-09

**Authors:** Pongsakorn Poovarodom, Chaiy Rungsiyakull, Jarupol Suriyawanakul, Qing Li, Keiichi Sasaki, Nobuhiro Yoda, Pimduen Rungsiyakull

**Affiliations:** 1 Department of Prosthodontics, Faculty of Dentistry, Chiang Mai University, Chiang Mai, 50200 Thailand; 2 Digital Laboratory Innovation, Department of Reconstructive and Rehabilitation Sciences, James B. Edwards College of Dental Medicine, Medical University of South Carolina, Charleston, SC 29425 USA; 3 Department of Mechanical Engineering, Faculty of Engineering, Chiang Mai University, Chiang Mai, 50200 Thailand; 4 Department of Mechanical Engineering, Faculty of Engineering, Khon Kaen University, Khon Kaen, 40000 Thailand; 5 Faculty of Engineering, School of Aerospace, Mechanical and Mechatronic Engineering, The University of Sydney, Sydney, NSW 2037 Australia; 6 Miyagi University, Taiwa, Miyagi 981-3298 Japan; 7 Division of Prosthetic Dentistry, Graduate School of Dentistry, Tohoku University, Sendai, Miyagi 980-8576 Japan

**Keywords:** Optimization, Bone remodeling, Dental implant, Customized abutment, Finite element analysis, Dentistry, Computational models

## Abstract

The optimal configuration of a customized implant abutment is crucial for bone remodeling and is influenced by various design parameters. This study introduces an optimization process for designing two-piece zirconia dental implant abutments. The aim is to enhance bone remodeling, increase bone density in the peri-implant region, and reduce the risk of late implant failure. A 12-month bone remodeling algorithm subroutine in finite element analysis to optimize three parameters: implant placement depth, abutment taper degree, and gingival height of the titanium base abutment. The response surface analysis shows that implant placement depth and gingival height significantly impact bone density and uniformity. The taper degree has a smaller effect on bone remodeling. The optimization identified optimal values of 1.5 mm for depth, 35° for taper, and 0.5 mm for gingival height. The optimum model significantly increased cortical bone density from 1.2 to 1.937 g/cm^3^ in 2 months, while the original model reached 1.91 g/cm^3^ in 11 months. The standard deviation of density showed more uniform bone apposition, with the optimum model showing values 2 to 6 times lower than the original over 12 months. The cancellous bone showed a similar trend. In conclusion, the depth and taper have a significant effect on bone remodeling. This optimized model significantly improves bone density uniformity.

## Introduction

Late implant failures, which typically occurring at 5–10 years and associated with substantial bone loss^1^, are reported in 2.1% to 11.3% of cases^2,3^. These failures are often due to osseointegration breakdown, primarily caused by excessive biting forces^4–6^. Research using finite element analysis (FEA)^7,8^ suggests that occlusal loads concentrate at the implant’s marginal bone, which remodels in response to strain^9^, potentially causing microfractures and bone loss^10,11^. A recent systematic review provided evidence linking excessive occlusal forces to marginal bone loss around implants^12^. To mitigate such failures, assessing patient occlusion and designing prosthetics to evenly distribute forces is essential. Dental implant abutments significantly influence the direction and magnitude of forces applied to implants^13,14^.

Advancements in digital dentistry and CAD/CAM technologies now allow for custom abutment designs based on patient-specific implant axes, peri-implant tissue, and restorative space^15^. Zirconia, used as an alternative to titanium^16^, offers superior mechanical properties^17^ and supports cell adhesion while reducing bacterial adherence^18^. Hybrid zirconia abutments with titanium bases have been developed to address issues such as micro-movements at the implant-abutment interface^19^. However, there is limited scientific information on the outcomes of custom two-piece zirconia abutments^20^.

This study aims to formulate a novel design framework to optimize custom two-piece zirconia abutment parameters using a multi-objective optimization approach with bone remodeling algorithm subroutine in FEA^21^. The goal is to maximize and harmonize bone density in the peri-implant region. Key design parameters include implant placement depth^22–27^, abutment taper degree^28–30^, and gingival height (GH) of the titanium base^28–30^.

Varies implant placement depth is widely used to conserve peri-implant bone levels and prevent crestal bone loss^23,31,32^. In vivo and in silico studies highlight its benefits for bone remodeling, stress reduction, and controlled bone resorption^23,25,26,33,34^. However, there is controversy regarding subcrestal (0.5–3 mm beneath the cortical bone crest) versus equicrestal implant placement position (level with the cortical bone crest), with some studies suggesting more alveolar bone loss with subcrestal placement^35–38^.

The implant abutment’s transmucosal configuration affects peri-implant soft tissues and crestal bone loss^39–41^. The macrogeometry and dimensions of a subcritical contour influence the emergence of peri-implant biological width and marginal bone remodeling^42^. Abutment profiles with wide emergence angles (> 30°) may lead to peri-implant tissue compression and bone loss^28,29^. The taper degree of the abutment also alters occlusal force distribution^13^, impacting bone remodeling.

Titanium base abutments, available in various gingival heights (0.5 to 2 mm), influence marginal bone loss^43–47^ and soft-tissue adaptation^46,47^. Taller gingival heights improve soft-tissue adaptation, reposition the crown-abutment junction, and reduce inflammation^48^. They also enhance the stability^49^ and fracture resistance of custom two-piece zirconia abutments^50–52^.

The aim of this study is to enhance bone remodeling, increase bone density in the peri-implant region, and reduce the risk of late implant failure. A 12-month bone remodeling algorithm subroutine in finite element analysis was employed to optimize three parameters: implant placement depth, abutment taper degree, and gingival height of the titanium base abutment.

## Materials and methods

### Geometry and finite element analysis

**Figure 1:**
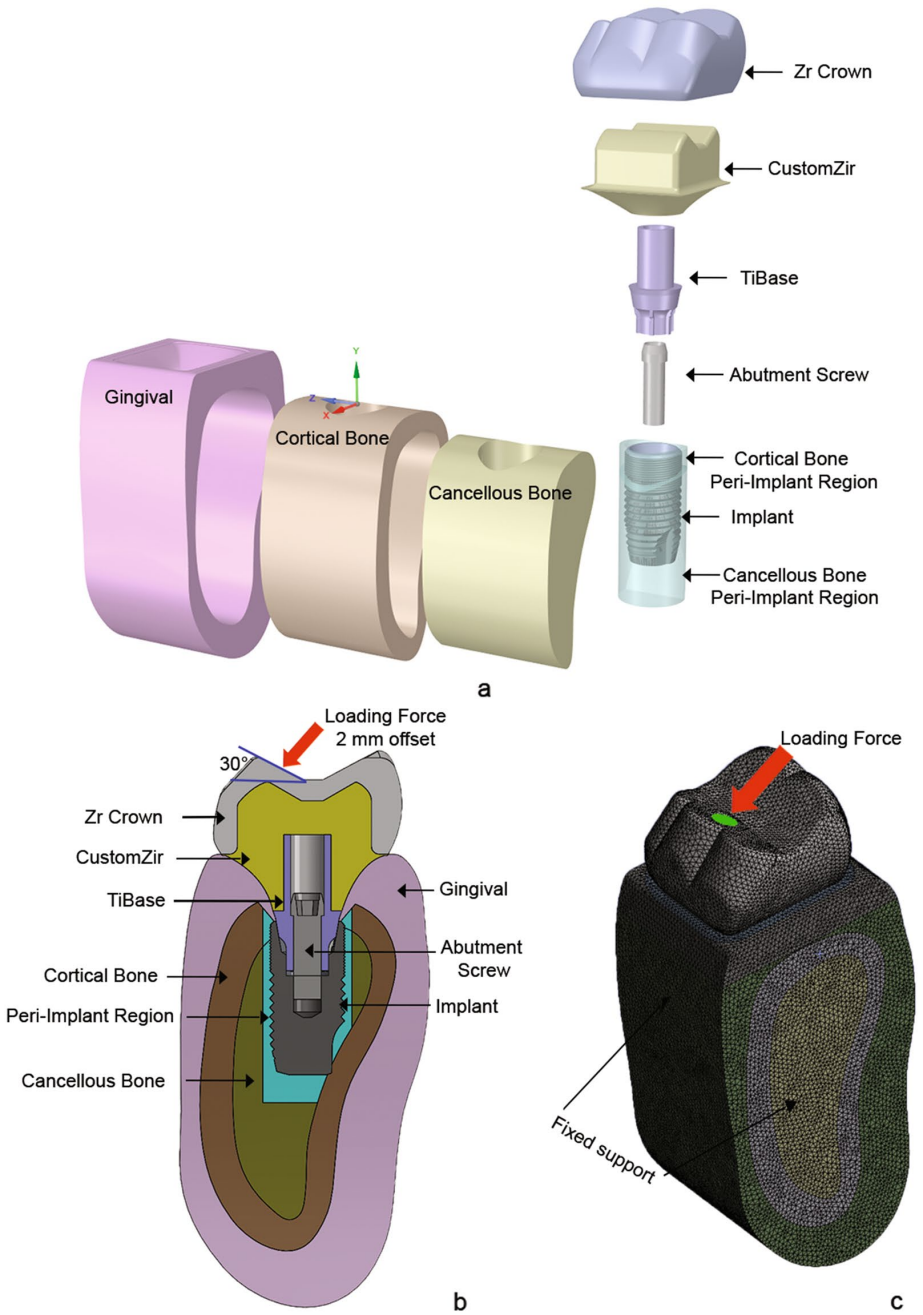
Model components and mesh model with simulation conditions: (**a**) exploded view displaying all components with labels; (**b**) bucco-lingual cross-section view of testing model with labeled components; (**c**) full isotropic view of finite element model with loading and boundary conditions.

**Figure 2:**
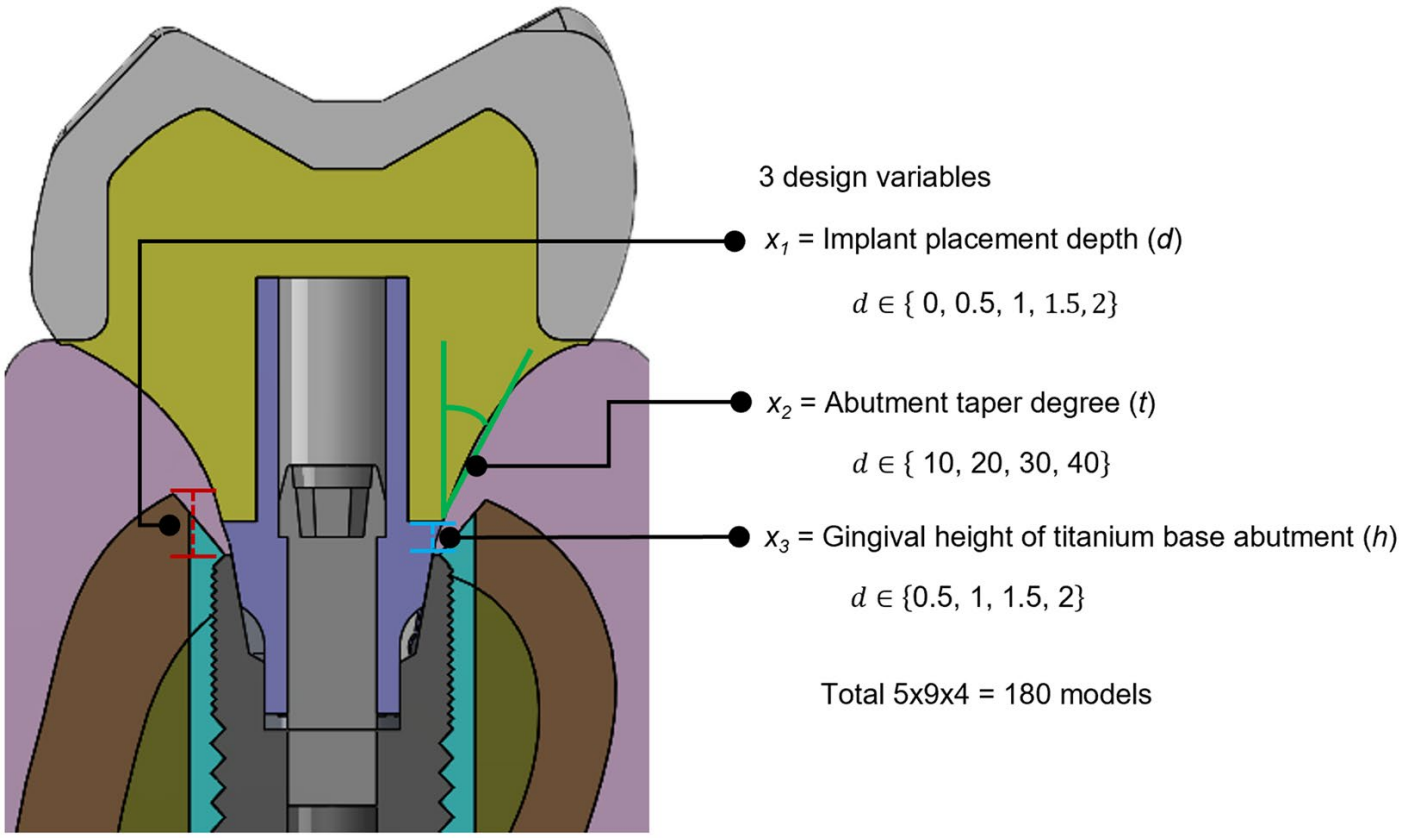
Bucco-lingual cross-section view of testing model and design parameters: *x*_1_ = implant placement depth (*d*), *x*_2_ = abutment taper degree (*t*), and *x*_3_ = gingival height of titanium base abutment (*h*).

The three-dimensional (3D) testing model consists of a dental implant, implant abutment, zirconia crown, abutment screw, and bone section (Fig. 1). A total of 180 3D models of different abutment designs based on 3 design factors were constructed, as shown in Fig. 2. The size of the dental implant used in the study was 5.4 mm in diameter and 11 mm in length, obtained from Astra Tech Implant System (OsseoSpeed™ EV; AstraTeech, Dentsply, York, PA, USA). A customized zirconia implant abutment on a titanium base abutment (TitaniumBase AT EV 5.4 GH1 L; AstraTech Dentsply, York, PA, USA) was selected. The abutment screw (Screw AT EV 5.4; AstraTech, Dentsply, York, PA, USA) was 7.6 mm in length, and the diameter of the screw shank was 2 mm. The zirconia restorative crown was 10 mm bucco-lingually, 12 mm mesio-distally, and 8 mm inter-occlusally. The implant, abutment and crown models were reconstructed using SolidWorks version 2017 (SolidWorks, SolidWorks Corp., Concord, MA, USA), while a section of the mandible bone in the molar area was created using cone beam computed tomography (CBCT) images, which were processed in Rhinoceros 3D version 4 (Robert McNeel and Associates, SEA, WA, USA). The bone sections were modeled with two segments: the outer shell, with an average thickness approximately 2 mm, represented the cortical bone, and an inner continuum core represented the cancellous bone, which was assumed to be perfectly bonded with the cortical layer ^53^. The peri-implant region, one millimeter around the implant, was segmented from the cortical and cancellous bone of each model to calculate the average volume of the results.

We applied a mechanical load of 200 N to the occlusal surface with a 2 mm horizontal offset from the center to the buccal side and an inclination from the vertical axis of 30°, which represents the normal biting force of an implant-supported single crown in the molar region^54^. Fixed support boundary conditions were applied at the mesial and distal sectional surfaces of the bone model, representing surrounding bone support. The implant, abutment, screw, and crown were assumed to have uniform and linear mechanical properties in terms of elasticity and isotropy^53,55,56^. For the sake of simplicity in the remodeling analysis, the hard tissues associated with these components were also assumed to be linearly elastic and isotropic^57^. To ensure accuracy and computational efficiency, a convergence study was conducted on the finite element (FE) mesh density of all components used in this study. The results indicated that an element size of 0.4 mm was appropriate. Specifically, 3D 10-node tetrahedral solid (SOLID187) elements were selected, with an average of 900,000 elements and 1,100,000 nodes incorporated into all models. Finally, finite element analysis was conducted in Ansys software version 2020 (Ansys, Canonsburg, PA, USA) on a workstation computer (13th Gen Core (TM) i9-13900K CPU 24 Core 3.0 GHz).

### Bone remodeling algorithm

In this study, we aimed to monitor and compare dynamic changes in bone density over a 12-month period. One cycle of the bone remodeling algorithm represents a 1-month time frame, derived from clinical data converted into the corresponding equation^58^. To achieve this, the average density volume and standard deviation values for the peri-implant bone region were collected through bone remodeling simulation using the bone remodeling algorithm. External and internal remodeling of bony structures can affect bone shape and density, according to bone remodeling theories^59^. Mechanical stimuli control the remodeling of bony tissues. Strain energy density (SED) is the most effective mechanical stimulation for long bone remodeling^60–62^. According to Frost, low mechanical stimulation from homeostatic levels prevents bone apposition or resorption, creating a ‘lazy zone’ of 50–1000 microstrain in bone remodeling. According to available literature, mechanical stimuli beyond 1000 microstrain can increase bone formation, and values above 2500 microstrain are considered physiological overloading. Conversely, the higher the mechanical stimulation, the faster the density changes, but this may not necessarily be true in clinical conditions where overloading can cause significant damage when the self-repair threshold of bony tissue is surpassed. As established in Refs.^63,64^, Eq. (1) can be changed to include a quadratic factor for overload-induced bone resorption:

1 $$ \Delta \rho = \left\{ {\begin{array}{*{20}l} {C_{1} \left[ {{\Xi } - K\left( {1 + s} \right)} \right]\Delta t - C_{2} [{\Xi } - K\left( {1 + s} \right)]^{2} \Delta t,} \hfill & {if{ }\,\Xi > K\left( {1 + s} \right)} \hfill \\ {0,} \hfill & {if\, K\left( {1 - s} \right) \le \Xi \le K\left( {1 + s} \right)} \hfill \\ {C_{1} \left[ {{\Xi } - K\left( {1 - s} \right)} \right]\Delta t,} \hfill & { if\, \Xi < K\left( {1 - s} \right)} \hfill \\ \end{array} } \right.. $$

The symbol “${\Xi }$” has been frequently utilized in various studies as a means to predict bone remodeling based on the mechanical stimulus per unit apparent density^63,61,65^, as defined by

2 $$ {\Xi } = \frac{U}{\rho }. $$

SED ($U$) (J/cm^3^) and local bone density ($\rho$) (g/cm^3^) determine “${\Xi }$” which is measured in J/g. Remodeling threshold tolerance ($s$) is 0.1. This equation calculates apparent density remodeling (∆ρ/∆t) by subtracting the mechanical stimulus from a bone remodeling reference value ^7^. For cortical and cancellous bone, C1 and C2 are 60 and 120 (month × g/cm^3^), respectively. K also equals 0.000036 J/g/cm^3^^58,66^. The time step (∆t) used in this study.

The cortical and cancellous bones are considered to have uniform density at the outset of the analysis, but subsequent remodeling could result in a more heterogeneous distribution of bone density in the spatial model^57,67^. This information is summarized in Table 1.

**Table 1:** Material properties of implant fixture, implant abutment, and crown materials.

Material/properties	Young’s modulus (GPa)	Poisson’s ratio	Density (kg/m^3^)
Titanium CP grade IV	110	0.3	4540
Zirconia (ZrO_2_)	200	0.35	2770
Gingiva	0.001	0.167	–
Cortical bone_Initial_	11.67	0.3	1400
Cancellous bone_Initial_	1.872	0.3	900

The relationship between the Young’s modulus $E$ (MPa) and the apparent density ρ (g/cm^3^) of peri-implant bone can be expressed as follows:

3 $$E = \left\{\begin{array}{c}2.349{\uprho }^{2.15} \,for \,cancellous\, bone \\ -21.93+24\rho\, for\, cortical \,bone \end{array}\right..$$

**Figure 3:**
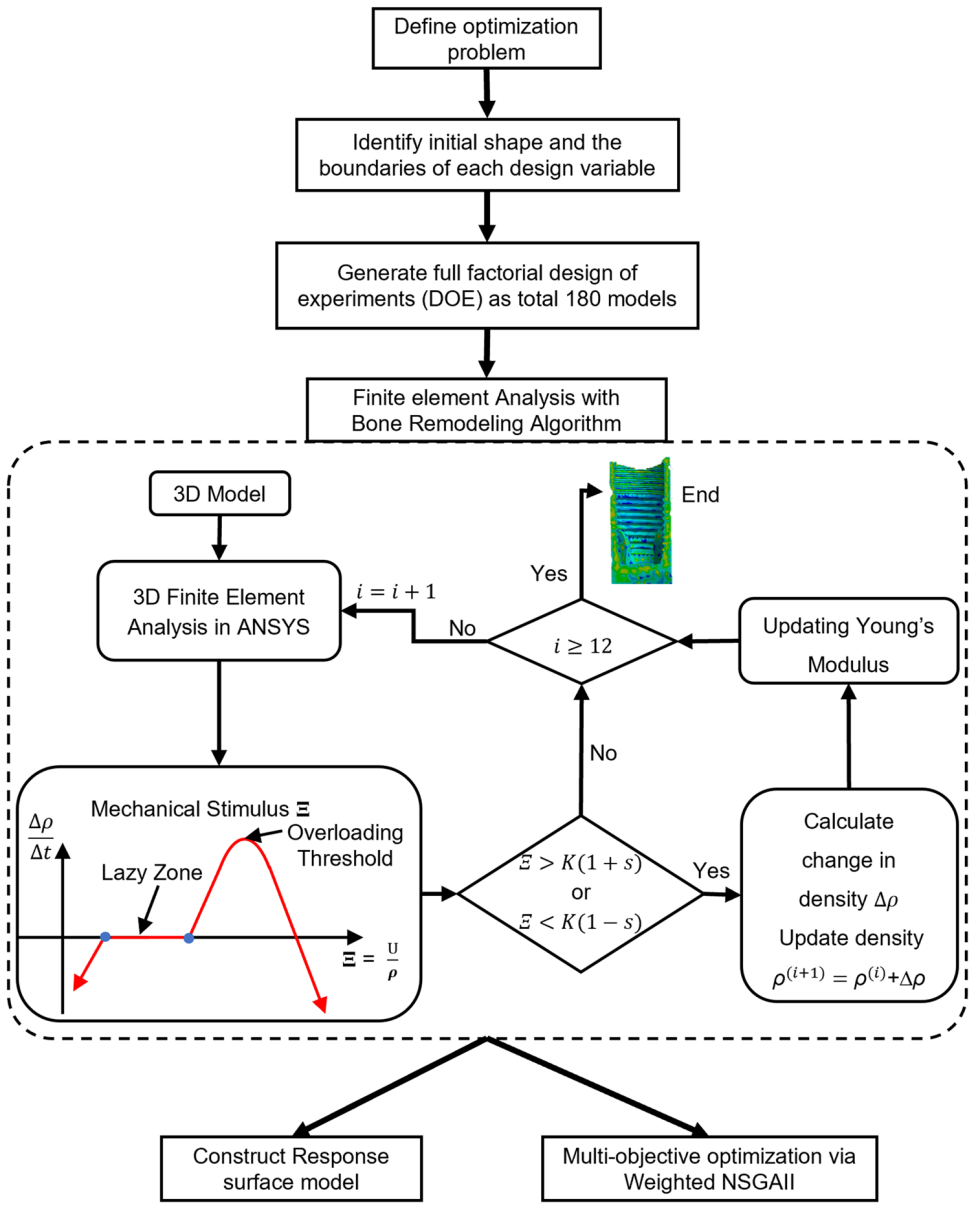
Flowchart of optimization process.

To simulate the bone remodeling process, a user-defined subroutine was used in Ansys. This subroutine evaluated and updated the material properties of each bone element according to the defined constitutive models for 12 months. The average density volume and standard deviation values for the peri-implant bone region were collected in all models for design analysis and optimization. The specific steps for the bone remodeling simulation are outlined in Fig. 3.

### Optimization problem

#### Objective functions and design variables

From the biomechanical point of view, it is expected that a customized implant abutment will enhance the process of osseointegration. This is because the transfer of load creates a specific alteration in the biomechanical environment in the area surrounding the implant, which sends mechanical signals that prompt the deposition of bone on the porous surface. To assess the level of osseointegration, the apparent density responses are computed by taking an average of the volume in a finite element model ^68^:

4 $$\overline{\rho }=\frac{1}{V}\underset{V}{\int }\rho dV\approx \frac{1}{{\sum }_{e=1}^{n}{V}_{e}} \sum_{e=1}^{n}{{\rho }_{e}V}_{e},$$

where ${V}_{e}$ denotes the volume of element *e, n* is the total number of elements in a selected region of interest and ${\rho }_{e}$ is the apparent density of element *e*. The remodeling rule utilized in this work implies that osseointegration and bone remodeling are improved and increased in relation to bone density.

The uniform enhancement of bone density is viewed as a secondary determinant of successful osseointegration. The homogeneity of increased bone density is gauged by a smaller standard deviation, signifying more consistent apparent density. The metric utilized to assess bone density uniformity within a specified area is standard deviation (σ), which is essentially the fluctuation in predicted bone density for each constituent element. The definition of standard deviation (σ) is as follows:

5 $$\sigma =\sqrt{\frac{1}{n} \sum_{e=1}^{n}{{(\rho }_{e}-\overline{\rho })}^{2}}.$$

To tackle the design optimization of a customized abutment, the implant placement depth (*d*), abutment taper degree (*t*), and gingival height (*h*) of the titanium base abutment are taken as the design variables, where x = (*d,t,h*)^T^. As a result, the design problem can be formulated in terms of apparent density (Eq. 6) and standard deviation (Eq. 7) as follows:

6 $$max{f}_{\rho }\left(x\right)=\overline{\rho }\left(d,t,h\right)=\frac{1}{V}\underset{V}{\int }\rho dV,$$

7 $$min{f}_{\sigma }\left(x\right)=\sigma \left(d,t,h\right)=\sqrt{\frac{1}{n} \sum_{e=1}^{n}{{(\rho }_{e}-\overline{\rho })}^{2}}.$$

The objective of this study was to optimize the dynamic changes in bone density of cortical and cancellous bone over a 12-month period. Previous research indicated differences in the increasing trend between cortical and cancellous bone. Moreover, fluctuations in the increasing trend of density differed every month. Therefore, it is crucial to prioritize the monthly divergences that have a significant impact on the overall trend of increasing bone density in order to achieve the optimal design. The weight of each output was computed on the basis of the increasing magnitude of each bone type over a 12-month period, as presented in Table 2. In summary, the objective functions are defined as follows:

**Table 2:** Weights for average density volume and standard deviation (σ) of density over 12 months.

Month	Type			
	Cortical bone		Cancellous bone	
	Density	σ	Density	σ
1	0.1357	0.1357	0.07466	0.07466
2	0.06947	0.06947	0.09669	0.09669
3	0.01361	0.01361	0.02671	0.02671
4	0.00834	0.00834	0.01472	0.01472
5	0.00561	0.00561	0.00982	0.00982
6	0.00424	0.00424	0.00703	0.00703
7	0.00327	0.00327	0.00547	0.00547
8	0.00274	0.00274	0.00424	0.00424
9	0.00221	0.00221	0.00349	0.00349
10	0.00186	0.00186	0.00279	0.00279
11	0.00157	0.00157	0.00241	0.00241
12	0.00137	0.00137	0.00196	0.00196
Sum	0.25	0.25	0.25	0.25
Total	1			

Objective function:

8 $$\left\{\begin{array}{c}\\ f1= Maximize \left( \sum_{i=1}^{n=12}{{\overline{\rho }}_{cortical \left(i\right)}\times w}_{cortical \left(i\right)}\right) \\ f2= Maximize \left( \sum_{i=1}^{n=12}{{\overline{\rho }}_{cancellous \left(i\right)}\times w}_{cancellous \left(i\right)}\right) \\ f3= Minimize \left( \sum_{i=1}^{n=12}{{\sigma }_{cortical \left(i\right)}\times w}_{cortical \left(i\right)}\right) \\ f4= Minimize \left( \sum_{i=1}^{n=12}{{\sigma }_{cancellous \left(i\right)}\times w}_{cancellous \left(i\right)}\right) \end{array}\right..$$

Constraints:

9 $$ \left\{ {\begin{array}{*{20}l} {g{1 = }\Xi_{cortical} \le 0.1 {\text{ j/g}}} \hfill \\ {g2 = \Xi_{cancellous} \le 0.05 {\text{ j/g}}} \hfill \\ {g3 = \sigma_{{\text{v cortical}}} \le 125 {\text{ MPa}}} \hfill \\ {g4 = \sigma_{{\text{v cancellous}}} \le 20 {\text{ MPa}}} \hfill \\ \end{array} } \right.. $$

The terms *g*1 and *g*2 represent the maximum mechanical stimulation in cortical and cancellous bone, respectively, which is responsible for invoking the phenomenon of overload in bone. The yield strength of cortical and cancellous bone, designated as *g*3 and *g*4, is utilized as a benchmark to ensure that the stress levels at each design point of the bone model do not exceed allowable thresholds.

However, it is difficult to derive analytical formulas for the objective functions given in Eq. (8). To address this issue, we used response surface methodology (RSM) based on polynomials to estimate the objective functions defined in Eqs. (6) and (7) in simpler forms. This approach was adopted because it is difficult to come up with analytical formulas for objective functions ^69^.

#### Response surface

Response surface methodology (RSM) is a mathematical and statistical technique that aims to comprehend how experimental variables influence the response variable while minimizing the number of samples required. RSM is particularly useful for optimizing design problems that are highly mathematically complex or do not have an analytical expression for the design functions. By applying RSM to design optimization, it is possible to reduce the cost of analysis and gain insights from only a small number of experiments. The response surface optimization module of Ansys was used to create the surface. The response surface was explored to investigate the relationship linking the average density volume, the standard deviation of density, and the design variables.

In this approach, a complex function $f(\mathbf{x})$ is approximated numerically in terms of a series of simple basis functions as ^57,70^

10 $$\widetilde{f}\left(\mathbf{x}\right)= \sum_{j=1}^{N}{a}_{j}{\varphi }_{j}\left(\mathbf{x}\right),$$

where $f(\mathbf{x})$ represents a surrogate model to approximate the true function $f(\mathbf{x})$ and N denotes the number of the basis functions ${\varphi }_{j}\left(\mathbf{x}\right),\mathbf{x}\equiv {[d,h,t]}^{T}\in {R}^{n}$. In general, the selection of a basis function should ensure both convergence and adequate accuracy. To determine the unknown parameters $\mathbf{a}=({{a}_{1},{a}_{2}{,\dots ,a}_{N})}^{T}$ in Eq. (10), $M (M>N)$ sampling points ${\text{x}}^{\left(i\right)}(i= \text{1,2},.,\text{M})$ are needed. If the remodeling results for each sample point are obtained to form a response vector $\mathbf{f}=({{f}^{\left(1\right)},{f}^{\left(2\right)},\dots ,{f}^{(M)})}^{T}$, the typical least-square method can be applied to determine the unknown parameter vector a by minimizing.

11 $$E\left(\mathbf{a}\right)= \sum_{i= 1}^{M}{\left[{\mathbf{f}}^{({\varvec{i}})}-{\mathbf{a}}^{{\varvec{T}}}{\boldsymbol{\varphi }}_{{\varvec{i}}}({\mathbf{x}}^{\left({\varvec{i}}\right)})\right]}^{2},$$

thereby determining the unknown vector, $\mathbf{a}=({{a}_{1},{a}_{2}{,\dots ,a}_{N})}^{T}$, as

12 $$\mathbf{a}={\left({\Phi }^{T}\Phi \right)}^{-1}\left({\Phi }^{T}\mathbf{f}\right),$$

where the matrix $\Phi$ denotes the values of the basis functions evaluated at these *M* sampling points:

13 $$\Phi ={\left[\begin{array}{ccc}{\varphi }_{1}({\mathbf{x}}^{\left(1\right)})& \cdots & {\varphi }_{N}({\mathbf{x}}^{\left(1\right)})\\ \vdots & \ddots & \vdots \\ {\varphi }_{1}({\mathbf{x}}^{\left(M\right)})& \cdots & {\varphi }_{N}({\mathbf{x}}^{\left(M\right)})\end{array}\right]}_{M\times N}.$$

By substituting Eq. (12) into (10), the expression of $\widetilde{f}\left(\mathbf{x}\right)$ can be fully defined, which mathematically relates the objective functions to the design variables.

#### Optimization strategy

Multi-objective function optimization is a common problem in engineering and other fields where there are multiple conflicting objectives to be optimized simultaneously. Nondominated sorting genetic algorithm II (NSGA-II) is a popular approach for solving such problems. NSGA-II is effective at finding a diverse set of nondominated solutions, which can be useful for exploring the tradeoffs between conflicting objectives.

The main goal of this study was to prevent late implant failure of implant-supported single crowns by improving the density with a homogeneous increase in both cortical and cancellous bone after prosthesis delivery. To achieve this, we aimed to find an optimal solution by maximizing the density and minimizing the standard deviation of density of both types of bone using NSGA-II. The flowchart used in this study is shown in Fig. 3.

The structure was parameterized and set as three discrete parameters: implant placement depth (*d*), abutment taper degree (*t*), and gingival height of the titanium base abutment (*h*). The implant placement depth (*d*) is the vertical distance from the implant platform to the crest of the alveolar bone. The abutment taper degree (*t*) is the angle measured from the vertical axis of the zirconia material above the titanium base abutment. The gingival height of the titanium base abutment (*h*) refers to the vertical distance from the implant platform to the portion of the abutment that supports the zirconia above it (Fig. 2). The parameter range can be written as follows:

- *x*_1_ = Implant placement depth (*d*) $\text{d}\in \left\{\text{0, 0.5, 1, 1.5, 2}\right\} \text{ mm}$;
- *x*_2_ = Abutment taper degree (*t*) $\text{d}\in \left\{\text{0, 5, 10, 15, 20, 25, 30, 35, 40}\right\}$ degrees;
- *x*_3_ = Gingival height of titanium base abutment (*h*) $\text{d}\in \left\{\text{0.5, 1, 1.5, 2}\right\}$ mm.

In a typical multi-objective optimization problem, there exists a set of solutions that are superior to the remaining solutions in the search space when all objectives are considered but are inferior to other solutions in the space in one or more objectives. These solutions are known as Pareto-optimal or nondominated solutions^71^. The remaining solutions are known as dominated solutions. Since none of the solutions in the nondominated set is absolutely better than any other, any one of them is an acceptable solution. The choice of one solution over another requires problem knowledge and a number of problem-related factors.

## Results

**Figure 4:**
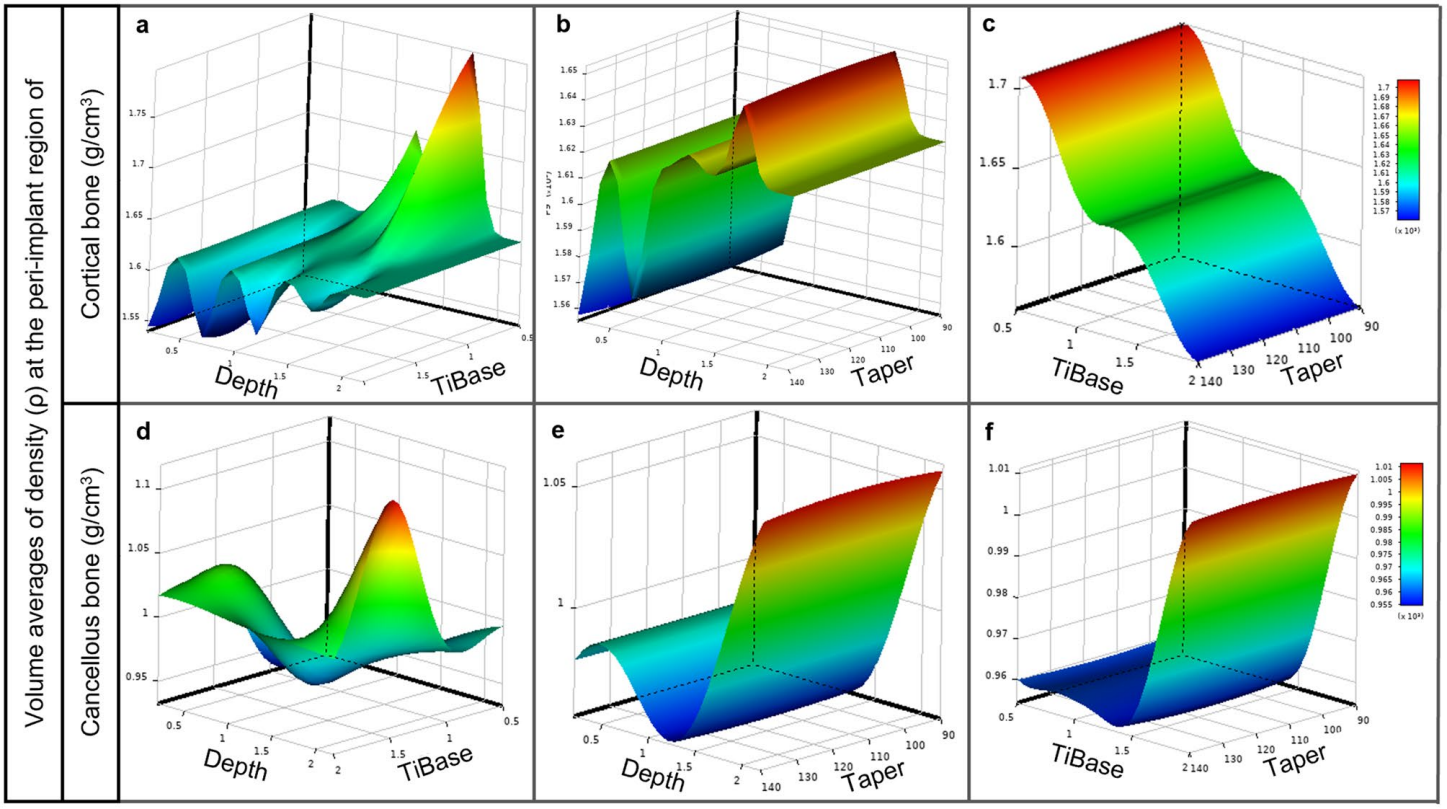
Response surface plots showing effects of implant placement depth (Depth), gingival height of titanium base (TiBase), and abutment taper degree (Taper) on average density volume in (**a–c**) cortical and (**d–f**) cancellous bone regions: (**a**) depth and TiBase in relation to cortical bone; (**b**) depth and Taper in relation to cortical bone; (**c**) TiBase and Taper in relation to cortical bone; (**d**) Depth and TiBase in relation to cancellous bone; (**e**) depth and Taper in relation to cancellous bone; (**f**) TiBase and Taper in relation to cancellous bone.

Figure 4 illustrates the impact of Depth, TiBase, and Taper on the average density volume in cortical and cancellous bone using response surfaces. Figure 4a,d show the correlation between Depth and TiBase in cortical and cancellous bone remodeling. In contrast, it is evident from Fig. 4b,c,e,f that the slope of the response surface refers to the degree of variation in relation to the *y*-axis, thereby demonstrating that Depth and TiBase have a greater effect on bone remodeling than Taper. Average density reaches the maximum at the largest Depth of 2 mm. However, in all 2 mm depth testing models, the overloaded mechanical stimulus triggered an algorithm that monitored the overloading element, resulting in damage to the peri-implant bone.

**Figure 5:**
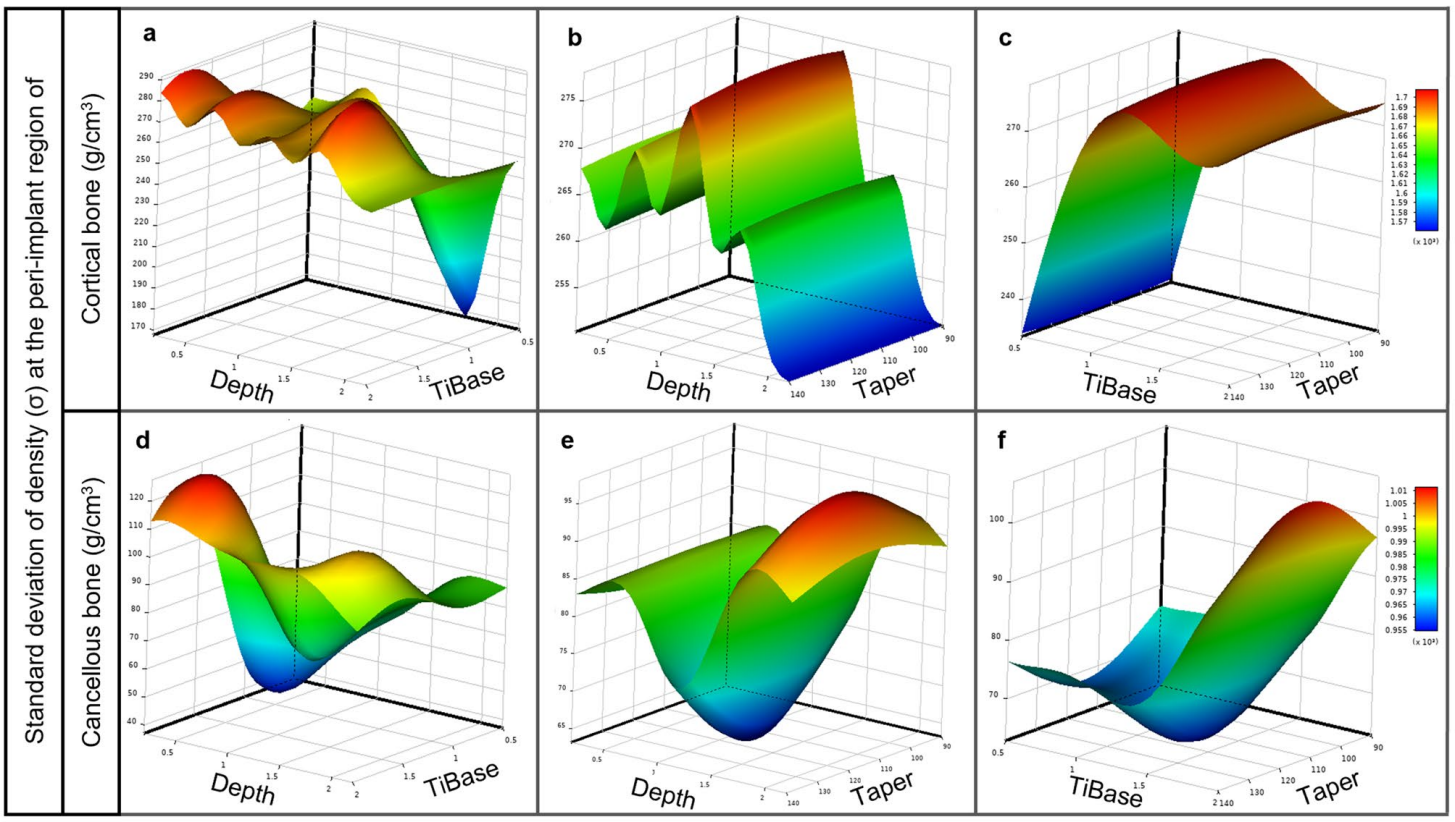
Response surface plots showing effects of implant placement depth (Depth), gingival height of titanium base (TiBase), and abutment taper degree (Taper) on standard deviation of density in (**a–c**) cortical and (**d–f**) cancellous bone regions: (**a**) depth and TiBase in relation to cortical bone; (**b**) depth and Taper in relation to cortical bone; (**c**) TiBase and Taper in relation to cortical bone; (**d**) depth and TiBase in relation to cancellous bone; **(e)** Depth and Taper in relation to cancellous bone; (**f**) TiBase and Taper in relation to cancellous bone.

Figure 5 illustrates the response surfaces showing the impact of Depth, TiBase, and Taper on the standard deviation of density in cortical and cancellous bone. It is important to note that, for cortical bone, Taper seems to have a comparatively smaller impact than other factors. This is evidenced by the relatively low gradient of the response surface along the *y*-axis, as depicted in Fig. 5b,c. Conversely, it can be seen that the correlation between Depth and TiBase displays fluctuations in its impact on the standard of deviation for cortical bone density, as illustrated in Fig. 5a. In contrast, it is observed that cancellous bone has a significant effect on the standard deviation of bone density depending on the three design variables. The fluctuation of the response surface is illustrated in Fig. 5d–f.

**Figure 6:**
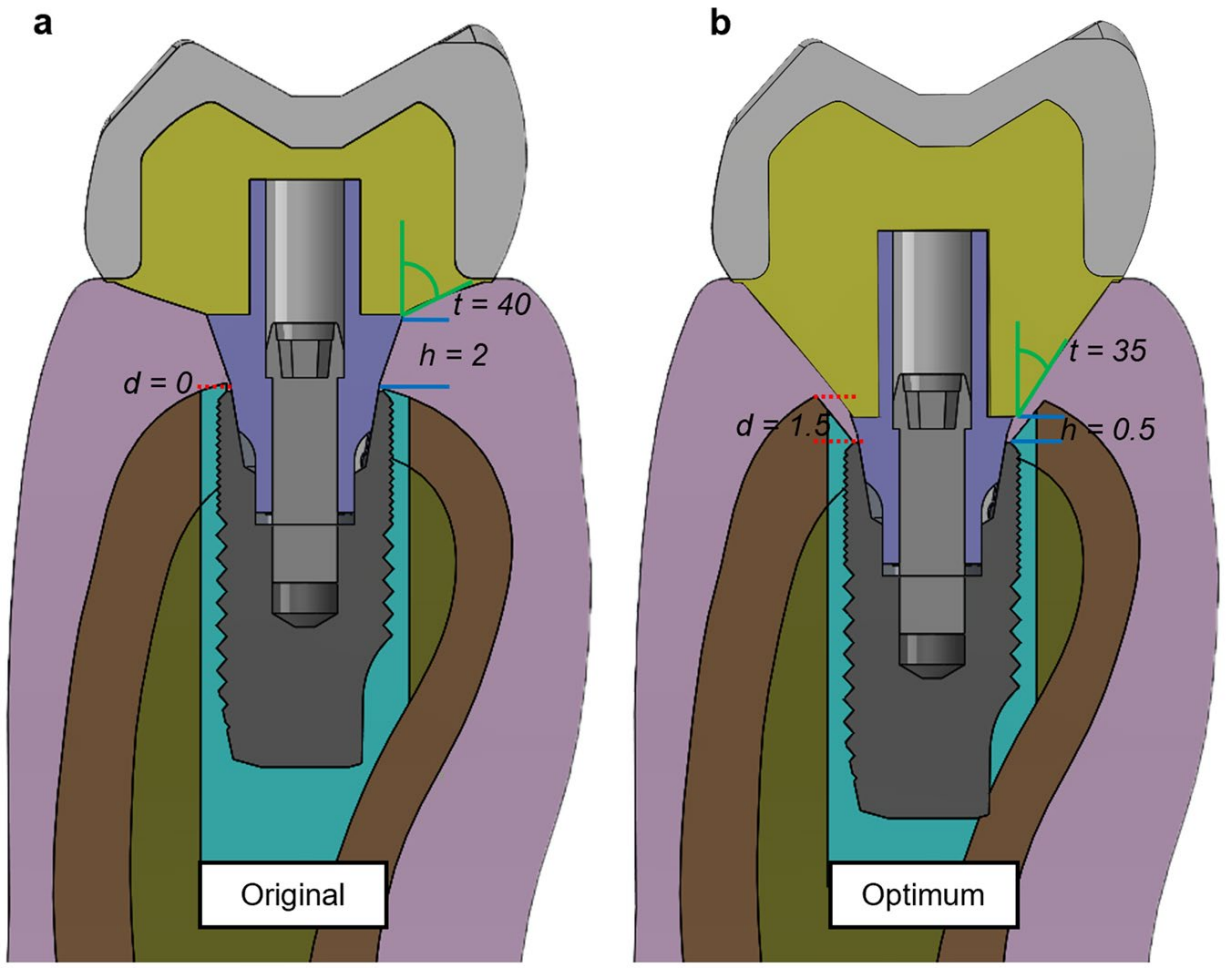
Comparison between (**a**) original model and (**b**) optimum model.

The results of the multi-objective optimization, aimed at maximizing average bone density volume while minimizing the standard deviation of bone density over a 12-month period, are presented and compared to the original model in Fig. 6. Notably, the original model (Fig. 6a) was developed according to the common restoration protocol for replacing a single tooth in the molar area. Typically, the dentist will position the implant at the same level as the marginal bone and choose a gingival height of the titanium base that matches the gingival margin. However, for this study, the gingival model was adjusted to be 2 mm from the marginal bone. Additionally, the taper degree, which is usually determined by the laboratory as a function of the width of the crown margin, was set to 40° in the current original model. The optimum design point values for implant placement depth (*d*), abutment taper degree (*t*), and gingival height of titanium base (*h*) were 1.5 mm, 35°, and 0.5 mm, respectively (Fig. 6b). Interestingly, the optimal design showed variations in all parameters compared to the original design. The depth of implant placement, for instance, increased from 0.5 to 1.5 mm. On the other hand, the gingival height of the titanium base decreased from 2 to 0.5 mm in the optimal design. Similarly, there was a slight reduction in taper degree, dropping from 40° to 35°.

**Figure 7:**
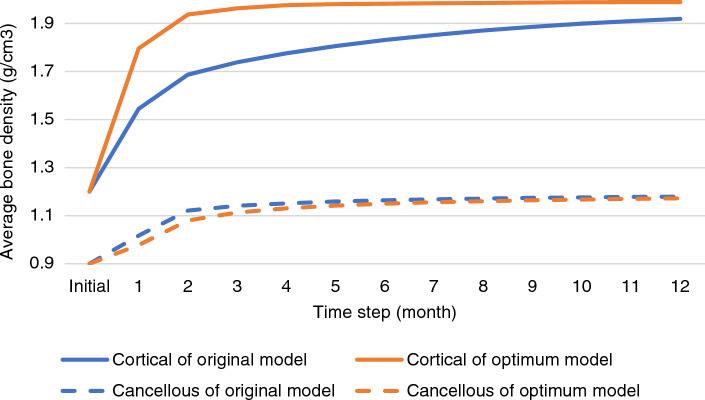
The volume averages of density (ρ) in the peri-implant regions of both cortical and cancellous bone over 12 months were compared between the original model (blue line) and the optimized model (orange line).

The average densities of the peri-implant regions of the cortical and cancellous bones are plotted in Fig. 7. The results for the cortical bone exhibited a dramatic increase in average density during the first 4 months, followed by a gradual rise until reaching the highest density at 12 months in each model. The optimum model significantly increased cortical bone density from 1.2 to 1.937 g/cm^3^ in 2 months and reached equilibrium in the 3rd month, while the original model increased density to 1.545 g/cm^3^ in 2 months and reached 1.91 g/cm^3^ in 11 months, achieving equilibrium in the 11th month. The cancellous bone showed a similar trend in both models. However, the original model exhibited a higher increase rate in cancellous bone density in the first 2 months. Afterward, the cancellous bone density of both the original and optimum models appeared to increase at a similar rate.

Table 3 presents a comparison of the objective functions between the original and optimized models across a 12-month span. Notably, the optimized model exhibits a significantly enhanced trend of average volume density in the cortical bone, coupled with a declining trend in the σ of cortical bone density. However, the trajectories of average volume density and σ of cancellous bone density appear to be slightly less favorable during the initial stages, specifically from the first to the sixth month. Following this period, the trends become more comparable between the original and optimized models.

**Table 3:**
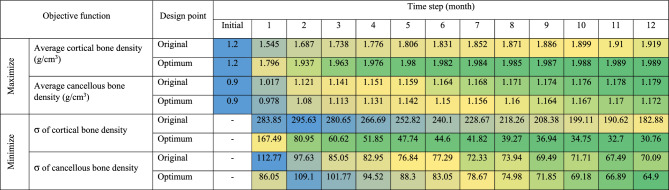
Objective function comparison between original and optimal models over 12 months (blue, yellow, and green indicate optimal values on a conditional color scale, which ranges from the upper to lower bound).

Table 4 shows a comparison of constraint conditions between the original and optimal models over 12 months. Notably, all constraint values remain within the prescribed upper limit. The optimized model generally exhibits higher constraint values across all parameters when compared to the original model, with the exception of the maximum mechanical stimulus of cancellous bone, for which the optimized model indicates a marginally lower value.

**Table 4:** Constraint conditions comparison between original and optimal models over 12 months.

Constraint condition	Design point	Timestep (month)											
		1	2	3	4	5	6	7	8	9	10	11	12
Maximum mechanical stimulus of cortical bone (J/g)	Original	0.040	0.071	0.071	0.070	0.070	0.070	0.070	0.070	0.070	0.070	0.070	0.070
	Optimum	0.052	0.096	0.095	0.095	0.095	0.095	0.095	0.094	0.094	0.094	0.094	0.094
Maximum mechanical stimulus of cancellous bone (J/g)	Original	0.024	0.047	0.044	0.044	0.049	0.044	0.048	0.045	0.048	0.045	0.044	0.044
	Optimum	0.020	0.037	0.033	0.033	0.033	0.033	0.033	0.033	0.033	0.033	0.033	0.033
Maximum von Mises stress of cortical bone (MPa)	Original	32.231	30.534	30.586	29.521	29.296	29.468	29.274	29.446	29.271	29.443	29.269	29.442
	Optimum	42.585	33.663	32.497	32.489	32.507	32.493	32.482	32.474	32.467	32.463	32.459	32.457
Maximum von Mises stress of cancellous bone (MPa)	Original	4.823	3.926	2.798	2.559	2.256	2.245	2.191	2.101	2.164	2.375	2.149	2.136
	Optimum	7.313	4.727	4.884	4.896	4.907	4.915	4.920	4.924	4.927	4.930	4.932	4.934

## Discussion

This study presents a novel approach to optimizing the design of two-piece zirconia custom abutments using a multi-objective optimization framework. By integrating a 12-month bone remodeling algorithm with FEA, we simultaneously optimized implant placement depth, abutment taper degree, and gingival height of the titanium base abutment. This innovative methodology not only enhances the biomechanical performance of dental implants but also provides personalized treatment guidelines based on patient-specific parameters. These advancements represent a significant contribution to dental implantology and precision medicine.

### Response surface

From the response surface obtained (Figs. 4, 5), it is evident that the implant placement depth (*d*) and gingival height of the titanium base abutment (*h*) are crucial factors in bone remodeling, whereas the abutment taper degree (*t*) does not significantly impact bone remodeling. This is supported by the steepness of the response surface, as shown in Fig. 4b,c,e,f, which indicates the rate of change with respect to the *y*-axis (Depth and TiBase). In Fig. 5b,c,e,f, the abutment taper degree (*t*) appears to have a minimal impact on the standard deviation of bone density. Therefore, in the protocol for implant placement, depth (*d*) and gingival height of the titanium base abutment (*h*) can be taken as key factors for the restoration of single implants.

### Optimization calculation

This study carried out multi-objective optimization for a period of 12 months with the dual aims of maximizing average bone density and minimizing its standard deviation (Table 3). The optimized model derived from this study deviates from the original model that is commonly used by dentists for routine restorations. In the optimized model, the implant placement depth was shown to be at a 1.5 mm subcrestal position (Fig. 6b), which is deeper than the 0 mm or crestal position used in the original model (Fig. 6a). This altered placement led to the elimination of peri-implant bone above the implant platform, necessitating drilling to enable placement of the implant below the crestal bone. Despite this seeming tradeoff, an increase in peri-implant gingival volume around the implant abutment above the platform was ultimately observed. In this subcrestal position, we noted a significant rise in the density apposition of the cortical bone, along with a more uniform increase in density, which was considerably higher than in the original model. Notably, these changes occurred without any risk of over-constraints (Table 4). Clinical studies have shown that subcrestal placement of implants can prevent marginal bone loss, supporting our findings. This placement aids in maintaining peri-implant bone levels and minimizes the risk of crestal bone resorption^23,25,26,33,34,72^. However, it is essential to conduct further studies on the formation of a new biological width with an implant in a subcrestal position restored by custom abutment. This will aid in better comprehending potential biological risks in the future.

In the optimized model, the gingival height of the titanium base had the lowest design boundary value at 0.5 mm. This differs from the usual clinical selection protocol, according to which the dentist typically chooses the gingival height of the abutment to match the distance from the implant platform to the gingival margin. In this study, this distance was equal to 2 mm in the original model. On the basis of the response surface results (Fig. 5a,c,d,f), the gingival height of the titanium base appears to contribute to minimizing the standard deviation (SD) of both cortical and cancellous bone density. However, the reduced gingival height of the titanium base could result in a thinner custom zirconia abutment connector above it, which could lead to the mechanical risk of abutment fracture^52^. Moreover, the junction between the zirconia and titanium abutments may be located beneath the gingiva, which could potentially irritate the gingiva if any cement residue is present^73^. Our findings suggest a direct relationship between the implant placement position and the gingival height of the titanium base in controlling the taper degree of the abutment. In scenarios where the implant placement depth is level with the bone (0 mm) and the gingival height of the titanium base abutment is 2 mm, as in the control model, the available dimensions for creating the abutment taper are relatively limited. In contrast, in the optimal model, where the implant is placed at a 1.5 mm subcrestal position and restored with a titanium base abutment at 0.5 mm gingival height, there is considerably more space to create a range of custom abutment shapes. Hence, the degree of the abutment taper is contingent on both the depth of the implant placement and the gingival height of the titanium base abutment. Furthermore, it was observed that the influence of the abutment taper on bone remodeling is relatively minimal.

On the basis of the findings, it was determined that the taper of the abutment does not have a significant impact on bone remodeling, as demonstrated by the response surface in Fig. 4. As shown in Fig. 4, Depth and TiBase had a greater influence on the response surface than Taper, resulting in a steep curve that indicates the rate of change with respect to either one. Nevertheless, various clinical studies have established a correlation between abutment taper and marginal bone loss^39–41,74^. However, it is important to consider other factors that may influence this relationship, such as the shape of the abutment and its effect on peri-gingival architecture and blood flow. Therefore, a narrower abutment may be preferable, as it allows more room for the peri-implant gingiva to wrap around it, acting as an elastic band to prevent micro-pathologies that can lead to peri-implant bone loss caused by infection or inflammation^75–77^. However, when designing a narrow abutment, it is important to consider the strength of the zirconia abutment and the manufacturer’s capabilities^52,78^. Additionally, the analysis in this study was based on isotropic and bonded conditions, which may not fully capture the behavior of nonlinear materials such as human gingival tissue. Therefore, further research on gingival tissue and abutment design is necessary to clarify this relationship.

### Design optimization

In this study, we employed a full factorial design of experiments (DoE) to create testing models within the design space. The DoE was based on three variables: implant placement depth (*d* = 0, 0.5, 1, 1.5, and 2 mm), abutment taper degree (*t* = 0°, 5°, 10°, 15°, 20°, 25°, 30°, 35°, and 40°), and gingival height of the titanium base abutment (*h* = 0.5, 1.5, and 2.0 mm). These variables were chosen using discrete parameters derived from typical clinical implant placement protocols, dentist expertise, and a measurement scale of 0.5 mm provided by the implant insertion instrument. The gingival height differences for titanium base abutments were determined by considering data from manufacturers and commercially available implant systems. The range of abutment taper degrees was selected on the basis of commonly fabricated devices in dental laboratories. These design parameters were uniformly distributed across the entire design space, resulting in a total of 180 design points covering the entire range. Considering the complexity of simulating bone remodeling in this study, it was essential to perform a comprehensive DoE to ensure accurate results within the design space. However, it is worth noting that the simulation and storage of results required a significant amount of time, which is a drawback of this protocol.

The optimal outcome of this study was obtained utilizing a weighted sum optimization process, in which equal weighting was assigned to the objective functions related to cortical and cancellous bone. Nonetheless, it is crucial to acknowledge that, in the clinical context, dentists must exercise their judgment on the basis of various aspects, including the initial bone quality and the specific conditions of individual patients. Therefore, while the findings of this study provide a valuable foundation, clinicians are urged to consider the unique circumstances of each patient when formulating a treatment strategy.

### Limitation

This study has certain limitations that need to be acknowledged. First, it relied on in silico analysis, therefore, it is necessary to validate the findings with subsequent clinical investigations. The initial bone density values, and the thickness of the cortical and cancellous bone, were based on a previous study, allowing for comparisons with similar bone density scenarios. For simplification and practical purposes, we assumed bone isotropy, which means we considered bone material properties to be uniform in all directions. This assumption may not fully capture the anisotropic nature of bone, which has different mechanical properties in different directions. Furthermore, model simplifications, bite force assumptions, variability in bone contact mechanics, and specific implant designs and abutment connections used in our study may not reflect the full range of clinical scenarios. These factors highlight the importance of further research and clinical validation to ensure the robustness and generalizability of our findings. However, further clinical investigation is needed to confirm the results in real-world scenarios. The Pareto fronts presented in this study only display one optimum solution. This limitation arises due to the sample size constraint and the discrete values of the design factors, which reflect real-world problems. Consequently, the optimization problem becomes less complex, resulting in only one solution for the output. It is important to note that the reported optimal depth in this study may be specific to the 3D shape and dimensions of the cortical and cancellous bone model used. These factors can vary among patients. Therefore, future research should delve deeper into the effects of implant depth and bone–implant contact in a more comprehensive manner.

Patient bite force capacity significantly influences dental implant outcomes. Variations in bite force affect the strain energy density (SED) distribution in the bone, impacting bone remodeling processes. Higher bite forces can enhance bone apposition but may risk overloading resorption, while lower bite forces may slow remodeling and affect implant stability. Understanding individual bite force capacities is essential for personalized treatment planning to optimize bone remodeling and implant success. Future studies should incorporate personalized bite force data for more accurate predictions.

## Conclusion

This research presents an innovative approach to enhancing the design of two-piece zirconia custom abutments in order to maximize bone remodeling and achieve a uniform increase in bone density. The findings of this study revealed the correlations between three key design factors and bone remodeling. It was observed that the taper degree of the abutment has a minimal impact on bone remodeling, while the taper degree is dependent on the depth of implant placement and the gingival height of the titanium base abutment. This study proposed an optimal design based on the research findings, indicating that implant placement at a depth of 1.5 mm accompanied by a gingival height of 0.5 mm for the titanium base abutment and an abutment taper of 35° comprise new clinical guidelines. These guidelines recommend subcrestal implant placement and restoration utilizing the lowest possible gingival height for the titanium abutment.

## Data Availability

The datasets generated and/or analyzed during the current study are not publicly available but are available from the corresponding author on reasonable request.
